# Impact of Biotic and Abiotic Stresses on the Competitive Ability of Multiple Herbicide Resistant Wild Oat (*Avena fatua*)

**DOI:** 10.1371/journal.pone.0064478

**Published:** 2013-05-16

**Authors:** Erik A. Lehnhoff, Barbara K. Keith, William E. Dyer, Fabian D. Menalled

**Affiliations:** 1 Department of Land Resources and Environmental Sciences, Montana State University, Bozeman, Montana, United States of America; 2 Department of Plant Sciences and Plant Pathology, Montana State University, Bozeman, Montana, United States of America; United States Department of Agriculture, United States of America

## Abstract

Ecological theory predicts that fitness costs of herbicide resistance should lead to the reduced relative abundance of resistant populations upon the cessation of herbicide use. This greenhouse research investigated the potential fitness costs of two multiple herbicide resistant (MHR) wild oat (*Avena fatua*) populations, an economically important weed that affects cereal and pulse crop production in the Northern Great Plains of North America. We compared the competitive ability of two MHR and two herbicide susceptible (HS) *A. fatua* populations along a gradient of biotic and abiotic stresses The biotic stress was imposed by three levels of wheat (*Triticum aestivum*) competition (0, 4, and 8 individuals pot^−1^) and an abiotic stress by three nitrogen (N) fertilization rates (0, 50 and 100 kg N ha^−1^). Data were analyzed with linear mixed-effects models and results showed that the biomass of all *A. fatua* populations decreased with increasing *T. aestivum* competition at all N rates. Similarly, *A. fatua* relative growth rate (RGR) decreased with increasing *T. aestivum* competition at the medium and high N rates but there was no response with 0 N. There were no differences between the levels of biomass or RGR of HS and MHR populations in response to *T. aestivum* competition. Overall, the results indicate that MHR does not confer growth-related fitness costs in these *A. fatua* populations, and that their relative abundance will not be diminished with respect to HS populations in the absence of herbicide treatment.

## Introduction

The reliance on herbicides for weed control has posed strong selection pressure for resistant populations, and there are now nearly 400 unique cases (plant species × site of action) of herbicide resistance in 217 plant species [1]. Most of these cases involve target site mutations that confer resistance to a single herbicide or related herbicides with the same mechanism of action. However, non-target-site-based resistance has recently become more common, and in some cases the use of one herbicide mode of action may substantially increase selection for non-target-site-based resistance genes that confer resistance to other unrelated herbicides [2], [3]. The physiological mechanisms of non-target-site-based resistance are usually based on enhanced herbicide metabolism or detoxification as mediated by cytochrome P450 monooxygenases [4] (hereafter P450s), glutathione *S*-transferases (GSTs) [5], and other enzymes of Phase II metabolism [6].

Ecological theory predicts that individuals with heritable resistance to an environmental stress may have an ecological disadvantage as compared to susceptible individuals in the absence of the stress [7], [8]. For example, herbicide resistant biotypes are predicted to experience a fitness cost as resources are shifted to resistance mechanism(s) rather than to growth and reproduction. Such fitness costs have been associated with a number of specific gene mutations conferring resistance to herbicides (see [9] for a review). For example, fitness costs have been demonstrated for the Pro197His and Trp547Leu mutations that confer resistance to acetolactate synthase (ALS)-inhibiting herbicides in prickly lettuce (*Lactuca serriola*) [10], [11] and Powell's amaranth (*Amaranthus powellii*) [12]. However, while Menchari et al. [13] determined fitness costs for the resistance-conferring Asp2078Gly and Ile2041Asn mutations in acetyl CoA carboxylase (ACCase) in slender meadow foxtail (*Alopecuris myosuroides*), they did not find fitness costs associated with the Ile1781Leu mutation. Similarly, Vila-Aiub et al. [9] found resistance costs for the Cys2088Arg mutation associated with resistance to ACCase herbicides in Wimmera ryegrass (*Lolium rigidum*), yet fitness costs were not demonstrated for the lle1781Leu mutation. Thus, particular target site mutations may or may not be associated with fitness costs in resistant populations.

Weed populations with herbicide resistance conferred by enhanced metabolic rates may be more likely to exhibit fitness costs, due to the constitutive and/or inducible overexpression of genes involved in energetically expensive pathways like those involving P450- and GST-mediated metabolism [14]. For example, a *L. rigidum* biotype (isolated from one population) with suspected P450-mediated herbicide metabolism produced less aboveground biomass and had a lower relative growth rate (RGR) [15] and was a weaker competitor with *T. aestivum* [4] in the greenhouse than susceptible biotypes. Similarly, Park and Mallory-Smith [16] found that plants from a metabolically-based resistant downy brome (*Bromus tectorum*) population produced less shoot biomass, leaf area and seeds, and was less competitive than plants from an adjacent susceptible population.

*A. fatua* is one of the most economically important weeds across the Northern Great Plains of North America, where it competes with and reduces yields of cereal and pulse crops [17], [18]. ACCase and ALS-inhibiting herbicides have been used to control *A. fatua* since the 1970s and 1980s, respectively. However, repeated use of these herbicides, as well as others such as triallate and difenzoquat, led to the evolution of herbicide resistant *A. fatua* populations in the 1980s and 1990s [19], [20], [21]. Recently, two *A. fatua* populations with resistance to multiple herbicides including triallate (emergence inhibitor), flucarbazone (ALS-inhibitor), imazamethabenz (ACCase-inhibitor), paraquat (membrane disruptor), and difenzoquat (membrane disruptor) were reported [22]. Resistance to some of these modes of action may be conferred by P450-enhanced metabolism [3], [23], [24] or by protection against oxidative stress via GST [6]. While controlling resistant weeds with herbicides is difficult, resistance via enhanced herbicide metabolism is especially problematic because weeds do not need to be exposed to a herbicide in order to be resistant to it [6].

Few studies have evaluated fitness costs for herbicide resistance in *A. fatua*. O'Donovan et al. [25] showed that shoot weight in triallate/difenzoquat-resistant *A. fatua* populations was generally greater than in susceptible ones, but this difference did not translate into competitive advantages. Similarly, Lehnhoff et al. [22] demonstrated that in non-competitive conditions, the MHR *A. fatua* populations utilized in this study had photosynthetic capacities and relative growth rates similar to susceptible populations, and while the MHR populations reached anthesis quicker, they ultimately produced fewer tillers and seeds.

Understanding the effects of herbicide resistance on individual fitness is crucial to the effective management of MHR weed biotypes. Specifically, assessment of fitness costs is important for the prediction of resistant biotype population demographics after the cessation of herbicide use [26], [27]. Three issues highlight the importance of assessing fitness costs of the MHR *A. fatua* populations. First, the observations that herbicide resistance fitness costs are not universal [9] indicates that each case should be examined individually. Second, the increasing incidence of MHR populations and their spread has profound implications for their prevention and management [28]. And finally, the fact that the MHR populations studied here are the first with confirmed constitutively elevated and inducible P450 expression levels (Keith et al. unpublished data) provides an important genetic resource in which to test the resource-based allocation theory.

The purpose of this research was to evaluate the competitive performance of two MHR and two herbicide susceptible (HS) *A. fatua* populations along a gradient of biotic and abiotic stresses. The biotic stress was provided by growing *A. fatua* plants in competition with three planting densities of *T. aestivum*, and the abiotic stress was a gradient of resource availability using three rates of N fertilization. Our objectives were to compare (1) total above-ground biomass production, and (2) relative growth rates of the HS and MHR *A. fatua* populations across a factorial combination of these abiotic and biotic gradients.

## Materials and Methods

### Plant Materials

Two herbicide susceptible (HS1 and HS2) *A. fatua* populations were used as controls. Population HS1 was obtained originally from seeds collected from a field adjacent to where MHR populations were collected, and subsequently confirmed to be 100% HS. All seeds were collected with private landowner permission, and no endangered or protected species were involved in this research. The second susceptible population (HS2, technically a biotype) was the nondormant inbred SH430 biotype used for seed dormancy research [29], [30]. This biotype exhibited similar growth to HS1 and the MHR populations in previous research [22].

MHR populations (MHR3 and MHR4) used for this research were derived from seeds collected from two *A. fatua* populations not controlled by 60 g a.i. ha^−1^ pinoxaden (Axial, Syngenta Crop Protection, Inc., Greensboro, NC, USA; ACCase inhibitor) from two fields in Teton County, Montana, USA in 2006. To ensure a 100% resistant population, these seeds (initially about 90% resistant to 60 g a.i. ha^−1^ pinoxaden) were subjected to two generations of recurrent group selection with 50 plants per generation by spraying with the same pinoxaden dose. Because we maintained some genetic diversity by using 50 random seeds in each generation, we refer to MHR3 and MHR4 as populations rather than biotypes. Therefore this work assessed the population level effects of MHR [22] and did not explicitly assess fitness costs of resistance as would be done via isogenic lines of MHR biotypes [31].

Prior to conducting this study, MHR3 and MH4 populations were determined to be resistant to field use rates of difenzoquat, flucarbazone, imazamethabenz, and tralkoxydim as compared to HS1 and HS2 [22]. Resistance to triallate and paraquat was subsequently determined for both MHR populations (Keith et al. unpublished data). We use the MHR acronym to describe these populations because they are resistant to members of five different mode of action families, and we suspect the presence of different physiological mechanisms (Keith et al. unpublished data).

### Plant Growth

This study was conducted as a complete randomized block design with four blocks of four *A. fatua* populations, three N fertilization rates, three levels of *T. aestivum* (Reeder hard red spring wheat) competition, and two harvest times, for a total of 288 experimental units (plastic pots, 17.8 cm dia. ×15.2 cm deep) per trial. It was conducted twice, simultaneously in two different greenhouses at the Montana State University Plant Growth Center under a 16-hr photoperiod of natural sunlight supplemented with mercury vapor lamps (165 μE m^−2^ sec^−1^) at 25±4 C. Pots were filled with a mixture of 1:1:1 [by vol] sphagnum moss, sand, and Sunshine Mix #1 (Sun Gro Horticulture, Inc., Bellevue, WA) and leached to reduce background N concentrations by draining four pot volumes of water through the soil over two days. Because of changes in greenhouse soil availability, different batches of soil were used in each greenhouse. Three soil samples, each composited from ten pots, were collected from each greenhouse and analyzed to determine initial concentrations of nitrate (NO_3_^−^), Olsen phosphorus (P), potassium (K^+^), and organic matter. The pots were then fertilized with ammonium sulfate at rates of 0, 50, or 100 kg N ha^−1^. In each pot, two seeds of one of the four *A. fatua* populations (HS1, HS2, MHR3, or MHR4) were planted 2 cm deep in the center of the pot, and *T. aestivum* seeds (0, 5, or 9) were planted evenly spaced around the circumference of the pot approximately 2 cm from the edge. *A. fatua* seedlings were thinned to the single largest individual per pot and *T. aestivum* seedlings were thinned to create densities of 0, 4, and 8 individuals per pot at 7 days after planting (DAP).

At 32 DAP, one half of the pots from each treatment were randomly chosen and *A. fatua* and *T. aestivum* plants were harvested separately by cutting them at the root crown, followed by drying at 40 C for 10 days, and weighing. The pots receiving 50 and 100 kg N ha^−1^ were then fertilized at these rates bi-weekly and weekly, respectively, for the next four weeks. The remaining plants were harvested at 61 DAP, and plant materials were dried and weighed as described above.

### Data Analysis

Differences in initial soil nutrient contents between greenhouse soil sources were evaluated by Student's t-test and were found to be different for concentrations of NO_3_^−^ (P<0.001), P (P = 0.038), and K^+^ (P = 0.019), but there were no differences in percent organic matter (P = 0.484) (Table 1). The effects of initial nutrient content on *A. fatua* biomass and RGR were evaluated via ANOVA with greenhouse as a random error term, and initial nutrient differences between greenhouses did not translate into differences in final *A. fatua* biomass (F_1,283_  = 1.09, P = 0.297) or relative growth rate (F_1,283_  = 3.03, P = 0.083), so data were combined for analysis.

**Table 1:** Soil nutrient concentrations.

Greenhouse	Nitrate (kg ha^−1^)	Phosphorus (mg kg^−1^)	Potassium (mg kg^−1^)	Organic Matter (%)
1	39.7±3.5^a^	13±0.0^a^	261.3±8.0^a^	7.2±0.7^a^
2	129.3±5.0^b^	14.7±0.6^b^	302.0±13.9^b^	7.6±0.6^a^

Initial nutrient and organic matter concentrations in greenhouse soil (mean ± SD, n = 3 per greenhouse) used in experiments to assess the impact of environmental and biological stressors on *Avena fatua* growth. Significant differences across greenhouses are indicated by different letters (P<0.05).

*A. fatua* RGRs were calculated following Hunt [32] (equation 1):
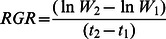
(1)where ln is the natural log, W_2_ and W_1_ are final and first harvest biomasses, respectively, and t_2_ and t_1_ are final and first harvest times in days. The biomass and RGR responses of the *A. fatua* populations to N rates and *T. aestivum* competition were analyzed via linear mixed effects-models, with N rate, *T. aestivum* biomass, and *A. fatua* population as fixed effects, and the greenhouse in which experiments were conducted as a random effect to account for initial soil differences, using the ‘lme4’ package in R (version 2.12.1, The R Foundation for Statistical Computing). Best models predicting *A. fatua* responses were determined based on the lowest Akaike Information Criterion (AIC) values. Change in AIC (ΔAIC) values between the best candidate model and all other models, and the models' AIC weights (*w*AIC, higher weight indicates better model), were also assessed for model comparison. For individual N rates, the response of each *A. fatua* population to *T. aestivum* was modeled via linear regression in R.

## Results and Discussion

**Figure 1:**
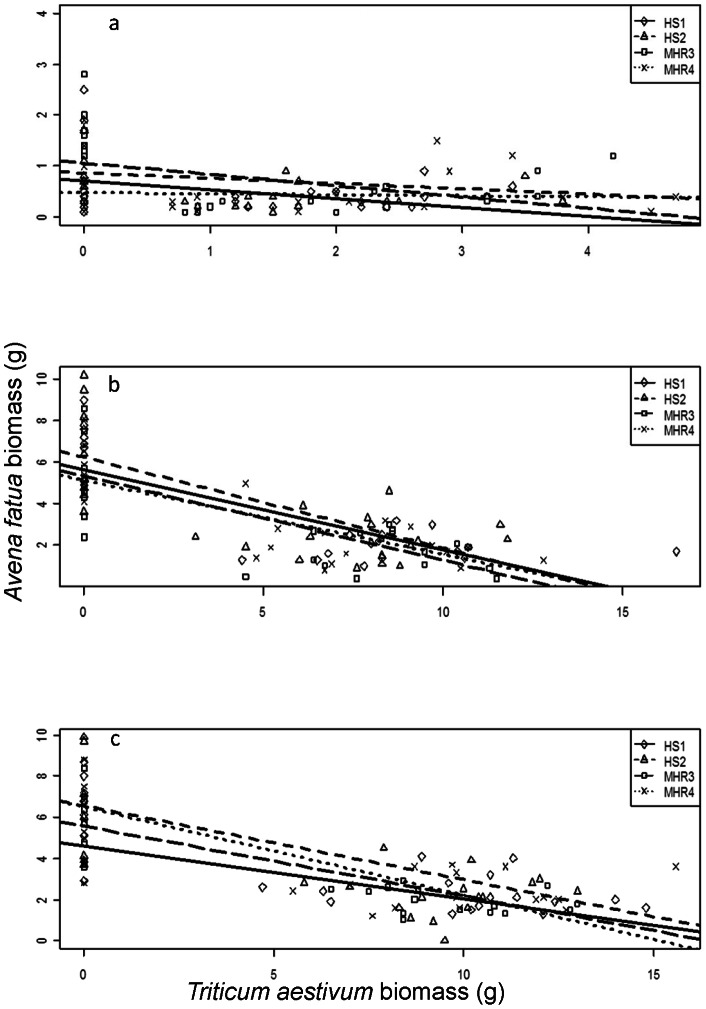
Effects of *Triticum aestivum* competition and nitrogen on *Avena fatua* biomass. Nitrogen fertilizer rates (N) are (a) 0, (b) 50 and (c) 100 kg N ha^−1^. HS1 and HS2 are herbicide susceptible *A. fatua* populations and MHR3 and MHR4 are multiple herbicide resistant populations. n = 8 for each N, *T. aestivum* competition, and *A. fatua* combination.

**Figure 2:**
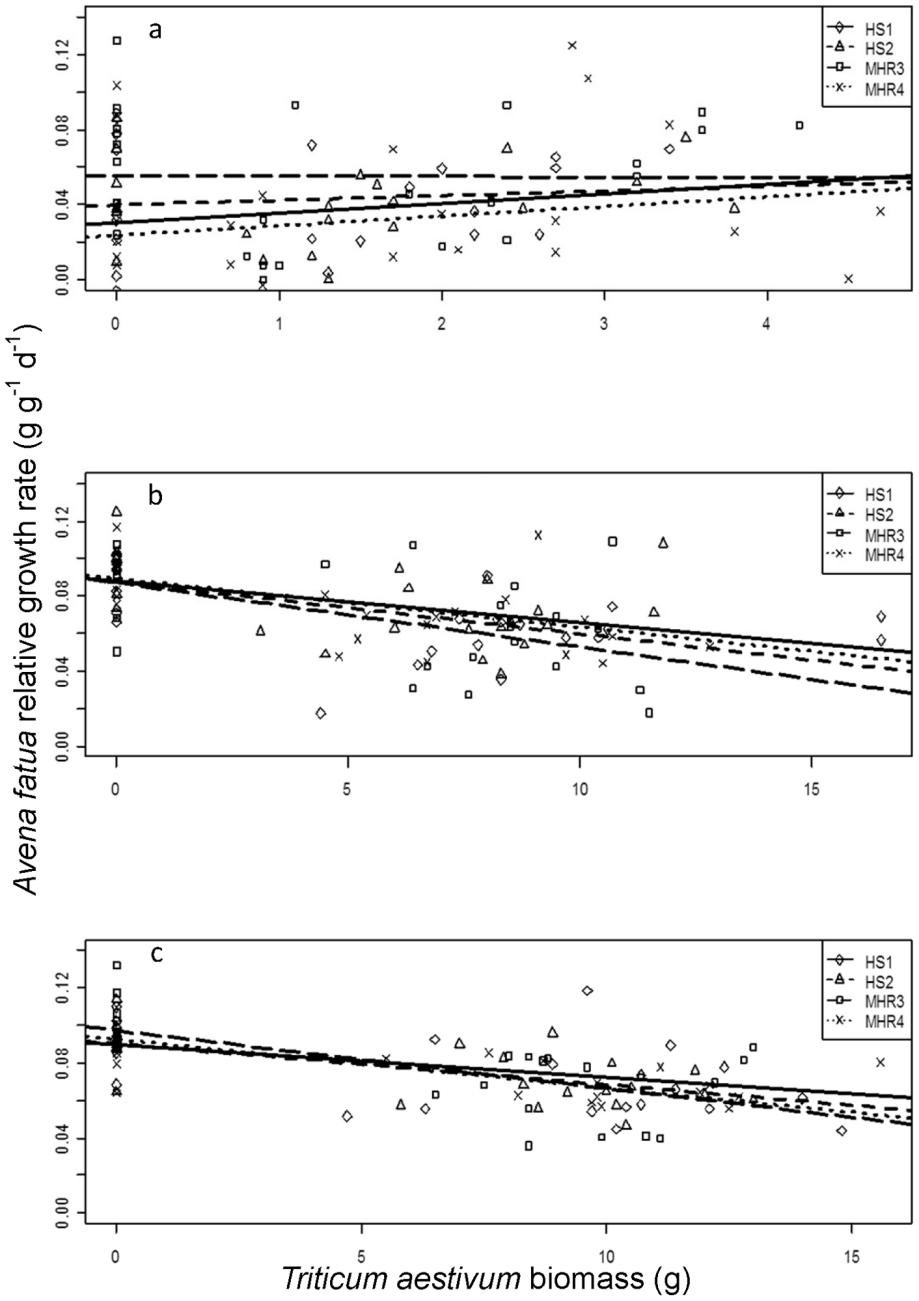
Effects of *Triticum aestivum* competition on *Avena fatua* relative growth rate. Nitrogen fertilizer rates (N) are (a) 0, (b) 50 and (c) 100 kg N ha^−1^. HS1 and HS2 are herbicide susceptible *A. fatua* populations and MHR3 and MHR4 are multiple herbicide resistant populations. n = 8 for each N, *T. aestivum* competition, and *A. fatua* combination.

The best candidate models predicting *A. fatua* biomass and RGR based on AIC scores and weights were N fertilization rate × *T. aestivum* biomass and N fertilization rate + *T. aestivum* biomass, respectively (Table 2). The second best candidate models for *A. fatua* biomass and RGR based on ΔAIC were N + *T. aestivum* biomass and N × *T. aestivum* biomass, respectively. The best models that included *A. fatua* population as a predictor had ΔAIC values of 22 and 14 for *A. fatua* biomass and RGR, respectively; however, these models had *w*(AIC) values of zero, indicating a poor fit to the data. At all N rates, the biomass of *A. fatua* decreased as *T. aestivum* biomass increased (P<0.05) (Figure 1) while the RGR of all *A. fatua* populations decreased with increasing *T. aestivum* biomass for the two higher N fertilization rates, but not at the zero N rate (P = 0.213) (Figure 2).

**Table 2:** Models predicting Avena fatua biomass and growth rate.

		Biomass			Relative growth rate		
Model variables	*k*	AIC	Δ (AIC)	*w*(AIC)	AIC	Δ (AIC)	*w*(AIC)
Intercept only	3	1296	237	0	−1158	62	0
N	4	1215	156	0	−1210	10	0.01
*T. aestivum* biomass	4	1287	228	0	−1143	77	0
*A. fatua* population	4	1300	241	0	−1145	75	0
N + *T. aestivum* biomass	5	1076	17	0	**−1220**	**0**	**0.97**
N + *A. fatua* population	5	1219	160	0	−1197	23	0
*T. aestivum* biomass + *A. fatua* population	5	1291	232	0	−1130	90	0
N × *T. aestivum* biomass	6	**1059**	**0**	**1**	−1212	8	0.02
N × *A. fatua* population	6	1231	172	0	−1177	43	0
*T. aestivum* biomass × *A. fatua* population	6	1298	239	0	−1114	106	0
N + *T. aestivum* biomass + *A. fatua* population	6	1081	22	0	−1206	14	0
N × *T. aestivum* biomass × *A. fatua* population	10	1096	37	0	−1139	81	0

Akaike information criterion (AIC) scores for mixed-effects models of *Avena fatua* biomass and relative growth rate, where *k* is the number of parameters predicted by intercept only, nitrogen fertilization rate (N), competition from *Triticum aestivum* (*T. aestivum* biomass), *A. fatua* population, and their interactions. Bold values indicate the best-fit models with the lowest AIC scores. Δ(AIC) is the change in AIC with respect to the best candidate model and *w*AIC is the AIC weight. N rate, *T. aestivum* biomass, and *A. fatua* population were fixed effects and greenhouse was a random effect included in every model. n = 8 for each N, *T. aestivum* competition, and *A. fatua* combination.

As expected, *A. fatua* biomass declined with increasing *T. aestivum* competition, although this relationship was not significant in the zero N treatments, where both *A. fatua* and *T. aestivum* biomasses were quite low compared to those in the 50 and 100 kg N ha^−1^ treatments. The minimal decrease of *A. fatua* biomass in the unfertilized pots can be explained by the apparent lack of competition from *T. aestivum*, as both species were stunted in these treatments (Lehnhoff, personal observation). The similar pattern observed with respect to *A. fatua* RGR can also be attributed to the low *T. aestivum* biomass in the unfertilized pots compared to fertilized ones. These results are generally in accordance with those reported by Blackshaw and Brandt [33] who showed that *A. fatua* competition with *T. aestivum* was unaffected by fertilizer rate.

More importantly, there were no differences in the response of HS and MHR populations to *T. aestivum* competition or N stress, suggesting that there were no growth-related fitness costs for multiple herbicide resistance. This is similar to our previous findings [22], where MHR populations did not experience growth-related fitness costs in the absence of competition. Because these MHR populations exhibit constitutively higher levels of P450 expression (Keith et al., unpublished data), the resource allocation theory would predict that the resulting resource diversion would be associated with a fitness cost. These results based on *A. fatua* growth therefore contrast with previous studies reporting various fitness costs associated with enhanced rates of herbicide metabolism [4], [15], [16].

The current results suggest that if competitiveness is positively related to reproductive fitness, the frequency of resistant alleles in the *A. fatua* MHR populations should not decline due to fitness costs. However, we previously showed that, in the absence of competition, one MHR population produced fewer seeds than a HS population in the greenhouse [22]. Thus, it is possible that if the selection pressure is interrupted, the proportion of MHR to HS seeds in the seedbank will decline, ultimately leading to a reduction in MHR populations, *sensu* Maxwell et al. [34] who predicted a decrease in resistant populations after the cessation of herbicide use. The relationship between *A. fatua* competitiveness (i.e., biomass production and RGR) and reproductive fitness is unknown, and will need to be examined under realistic field conditions to determine the actual implications for field management of MHR *A. fatua*.

While competitive ability provides a potential indication of the fitness costs associated with herbicide resistance [4], other traits that contribute to individual success through the entire life cycle, such as seed germination, seedling survival, and seed production should be included in a comprehensive analysis [14], [35]. In this context, this study provides useful information that can be integrated with future field research on MHR and HS demography. Such investigations will be required in order to develop successful alternative management options for herbicide resistant weed populations.

Finally, it should be noted that our study examined a small number of populations (two HS and two MHR). As Cousens et al. [36] discussed, a non-significant result from a small number of populations may not be enough to support a hypothesis that there is no fitness cost of resistance. Additional studies comparing the MHR populations to more HS populations could provide valuable insight into potential fitness costs. Alternatively, fitness costs could be assessed through the generation and testing of HS and MHR isogenic biotypes, and this process is currently underway in our laboratory.
